# Strategy toward Miniaturized, Self-out-Readable Resonant Cantilever and Integrated Electrostatic Microchannel Separator for Highly Sensitive Airborne Nanoparticle Detection

**DOI:** 10.3390/s19040901

**Published:** 2019-02-21

**Authors:** Maik Bertke, Jiushuai Xu, Michael Fahrbach, Andi Setiono, Hutomo Suryo Wasisto, Erwin Peiner

**Affiliations:** 1 Institute of Semiconductor Technology (IHT), Technische Universität Braunschweig, Hans-Sommer-Str. 66, 38106 Braunschweig, Germany; Jiushuai.Xu@tu-bs.de (J.X.); M.Fahrbach@tu-bs.de (M.F.); A.Setiono@tu-bs.de (A.S.); H.Wasisto@tu-bs.de (H.S.W.); E.Peiner@tu-bs.de (E.P.); 2 Laboratory for Emerging Nanometrology (LENA), Technische Universität Braunschweig, Langer Kamp 6a, 38106 Braunschweig, Germany

**Keywords:** nanoparticles, self-reading femtogram balance, cantilever resonator, FEM simulations, electrostatic particle collection

## Abstract

In this paper, a self-out-readable, miniaturized cantilever resonator for highly sensitive airborne nanoparticle (NP) detection is presented. The cantilever, which is operated in the fundamental in-plane resonance mode, is used as a microbalance with femtogram resolution. To maximize sensitivity and read-out signal amplitude of the piezo-resistive Wheatstone half bridge, the geometric parameters of the sensor design are optimized by finite element modelling (FEM). The electrical read-out of the cantilever movement is realized by piezo-resistive struts at the sides of the cantilever resonator that enable real-time tracking using a phase-locked loop (PLL) circuit. Cantilevers with minimum resonator mass of 1.72 ng and resonance frequency of ~440 kHz were fabricated, providing a theoretical sensitivity of 7.8 fg/Hz. In addition, for electrostatic NP collection, the cantilever has a negative-biased electrode located at its free end. Moreover, the counter-electrode surrounding the cantilever and a µ-channel, guiding the particle-laden air flow towards the cantilever, are integrated with the sensor chip. µ-channels and varying sampling voltages will also be used to accomplish particle separation for size-selective NP detection. To sum up, the presented airborne NP sensor is expected to demonstrate significant improvements in the field of handheld, micro-/nanoelectromechanical systems (M/NEMS)-based NP monitoring devices.

## 1. Introduction

Airborne nanoparticles (NPs) carrying toxic substances hold a great risk of adverse health effects on the human organism. The toxic effect of the particles depends strongly on their sizes, because particles with diameters less than 2.5 µm can enter the organism easily via the respiratory tract [1]. In particular, ultrafine particles (UFPs), which have diameters of <100 nm, are suspected to trigger alveolar inflammation and may lead to cardiovascular diseases [2]. The World Health Organization (WHO) considers indoor and outdoor air pollution as one of the largest environmental health risks [3]. Due to the increasing air pollution and use of UFPs in industry and consumer goods, there is a great need of small, highly sensitive and low-cost detector systems for real-time UFP monitoring. Optical sensors cannot detect UFPs due to their intrinsic properties of light diffraction limit and vanishing scattering cross-section [4]. Unlike optical-based sensing techniques, mechanical resonators can be used as micro-/nano-balances and their sensitivities are only limited by their mass, resonance frequency and intrinsic noise processes [5]. Therefore, various types of resonant micro/nanoelectromechanical systems (M/NEMS) were investigated as mass-sensitive airborne particle sensors to meet the demands, e.g., ultrasensitive doubly clamped nanoelectromechanical beam resonators [6,7], nanomechanical resonant filter-fiber [8], thermal-piezo-resistive SOI-MEMS oscillator based on a fully differential mechanically coupled resonator array [9], thermal-piezoresistive oscillator-based PM2.5 sensor with enhanced particle collection efficiency [10], and magnetic-excited, piezoelectric cantilever beam for particle detection [11]. Some of these resonators have very high resolutions, but are not suitable for handhold, low-cost sensor systems or a practical collection method is missing. The most commonly used method to collect particles on micro-/nano-resonators is by inertial impaction [8,9,10], which needs high velocity and yet very small particles rely on Brownian diffusion [8]. Bao et al. (2018) showed a method to trap micro-particles inside a cantilever beam using a micro-channel and micro-pillars [12]. However, particles <1 µm were not collected.

In previous works, we demonstrated airborne NP mass-concentration monitoring using a novel handheld device based on a silicon cantilever resonator and electrostatic particle collection [13,14]. Cantilever structures are used in many gravimetric measurement applications, e.g., electrochemical deposition processes [15], humidity sensing [16], gas sensing [17,18], and biosensors [19,20]. It enables a low resonator mass with low resonance frequency, which is very suitable for small devices due to a simple electrical system integration. Nevertheless, for the detection of low concentrations of UFPs, the resonator mass has to be miniaturized. Therefore, we achieved femtogram mass detection of single airborne NPs of 100 nm in diameter using vertical silicon nanowire resonators [21]. By combining it with a custom-built electrostatic aerosol sampler having a narrow and short path of airflow, the NP collection efficiency could be enhanced three times higher (up to 10.8%) than that employing commercial aerosol sampler (NAS TSI 3089) [22]. The electrode for particle collection is integrated on the resonator and electrostatic particle collection works at a low flow rate of 0.3 L/min of particle-laden air and a low particle velocity of ~1 m/s [14], i.e., does not need an external vacuum pump as required for particle impactors [23]. For portable/wearable applications, the small-size, low-weight, and fully integrated design together with low power and noiseless operation are clear advantages of the combination of resonant mass sensing with electrostatic particle sampling.

That result has demonstrated that besides the sensors, an effective and efficient sampler design needs to be developed. However, so far, the resonance frequency analysis of the nanowires has not been performed in real time in ambient air, but inside a scanning electron microscope (SEM), and could not be read out electrically, which is not practicable for a real application. Particle size separation based on electrostatic particle sampling has not been investigated so far, as the airflow channel provided in the previous designs was only a single path [14]. Therefore, here we consider an array of self-out-readable cantilevers integrated with micro-channels.

## 2. Sensor Concept

**Figure 1:**
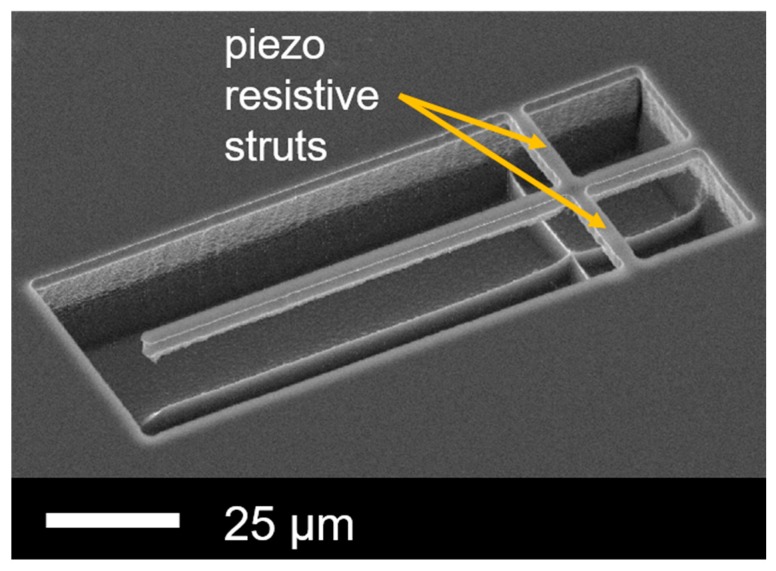
Etched Si micro cantilever with piezo-resistive struts.

**Figure 2:**
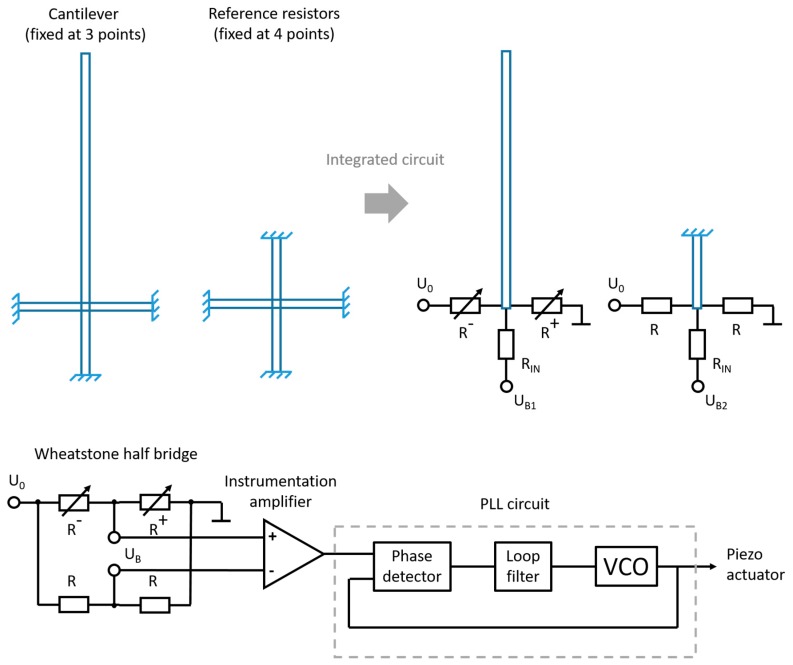
Electrical connections and circuit integration of the cantilever sensor.

M/NEMS resonators can work as a micro-/nano-balance, where small mass changes can be detected as resonance frequency (*f*_0_) shifts. The sensitivity of a resonant, gravimetric particle sensor can be estimated by

(1) $$\frac{\Delta f}{\Delta m}\approx \frac{f_{0}}{2m_{0}}$$

where *m* is the attached particle mass, *f* is the corresponding frequency shift, *m*_0_ and *f*_0_ are the resonator intrinsic mass and eigenfrequency, respectively [24]. Thus, to achieve high sensitivity, the resonator mass has to be very small. Furthermore, for a self-out-readable system, electrical integration is needed. Moreover, operation frequencies <1 MHz are proposed to avoid electromagnetic coupling effects and to simplify the development of the out-reading electronic circuitry. The proposed sensor design is based on a one-side-clamped, in-plane cantilever resonator as shown in Figure 1. This geometry has minimum structure mass at a low fundamental resonance frequency. The cantilever is laterally supported by piezo-resistive struts, which enables high-resolution real-time resonance tracking by a phase-locked loop (PLL) circuit [7,25,26,27]. For excitation, a piezo-electric die actuator underneath the sensor chip is used. Figure 2 illustrates the electrical connections and integrated circuit. The piezo-resistive struts of the cantilever and a fixed reference structure form a Wheatstone half bridge that can be read-out by an instrumentation amplifier. The measurement signal will be fed to the PLL circuit and coupled to a piezo actuator.

**Figure 3:**
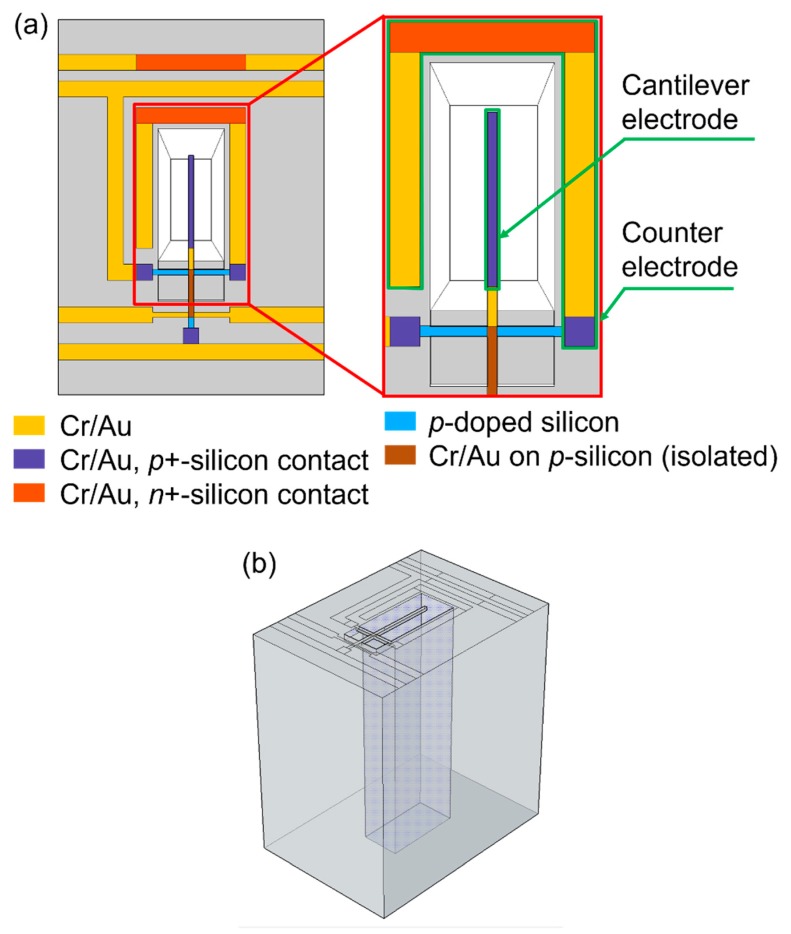
(**a**) Top and (**b**) axonometric schematics of the sensor design.

In our work, an electrostatic field is applied to achieve the particles collection on the cantilever surface. Therefore, an electrode on the cantilever and a counter electrode surrounding it are integrated, as illustrated in Figure 3a. Moreover, a micro-flow channel is added to the sensor chip (Figure 3b), allowing particles-loaded air to be directed to the cantilever and into the electrostatic field. The particle concentration measurements are performed in two separate steps: the particle collection phase, while particles are collected on the cantilever, and a sensing phase, while the resonance frequency is tracked by the PLL circuit. The collected particle mass is calculated from the resonance frequency shift between two consecutive measurement steps, which is taken to be proportional to the mass increase. After calibration against a stationary laboratory reference instrument (FMPS: Fast mobility particle sizer), airborne particle concentrations can be calculated [13,14].

## 3. Design Optimization by FEM

**Figure 4:**
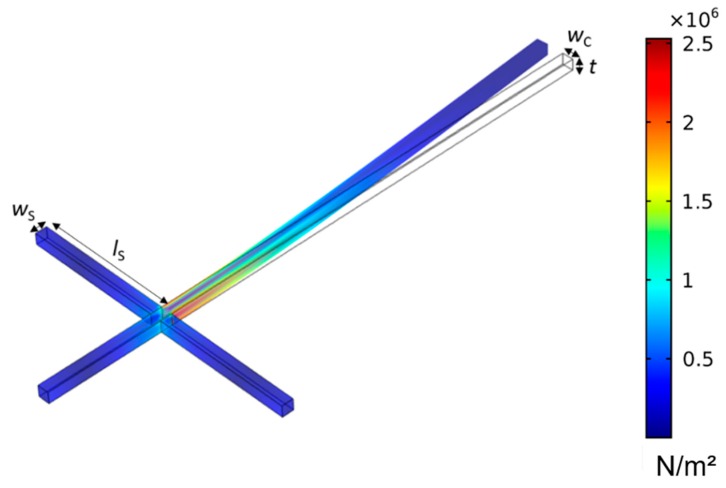
Cantilever deflection by FEM upon in-plane excitation with the generated von Mises stress marked by coloring.

Several finite-element modelling (FEM) simulations using COMSOL Multiphysics 4.4b were performed to optimize the sensor design parameters. One of the main aims was to obtain a maximum signal output of the sensors by maximizing the stress in the struts during in-plane excitation. Therefore, the structure width (cantilever width *w*_C_ and strut width *w*_S_) and structure thickness (cantilever thickness *t*_S_ and strut thickness *t*_S_) were scaled evenly with the constraints *w* = *w*_S_ = *w*_C_ and *t* = *t*_C_ = *t*_S_, respectively (Figure 4). A minimum structure size of (2–3) µm was chosen, so the necessary requirements for resolution and accuracy are fulfilled by conventional photolithography and mask alignment processes during manufacture. To keep *f*_0_ constant under the assumption of *f*_0_ ~*w*_C_/*l*_C_^2^ for a homogeneous rectangular cantilever, we tuned the cantilever length *l*_C_ = 100 µm by a factor of √(1 + *w*_C_/*w*_C_), while an initial *w*_C_ = 2 µm was increased by *w*_C_, resulting in a *f*_0_ ≈ 400 kHz. The material parameters are listed in Table 1.

**Figure 5:**
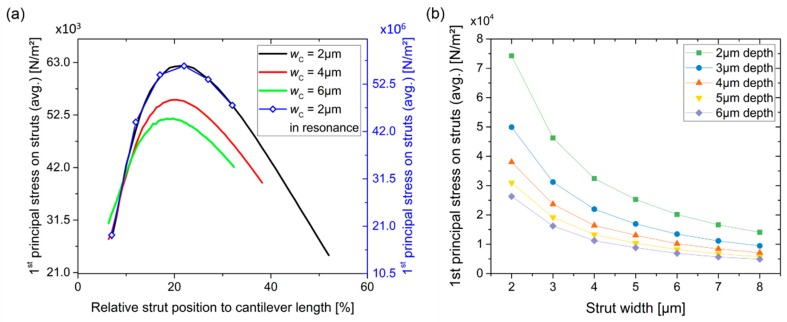
First principle stress on average over a piezo-resistive strut (**a**) vs. strut position along the cantilever length and (**b**) vs. strut width with strut/cantilever thickness as the parameter to the curves.

The optimum position of the struts *p*_S_ along the cantilever length *l*_C_ was determined for cantilevers with different widths (*w*_C_) in Figure 5a, using the first principal stress in the piezo-resistive struts. Therefore, we compared the stress *S* on the struts at dynamic base excitation in resonance in “Frequency Domain” of COMSOL Multiphysics, given by

(2) $$-\rho \omega^{2}u=\nabla \cdot S+F_{V}\exp\left(i\phi \right)$$

and a stationary body load *F*_V_ of 1.4 µN in “Stationary”, with

(3) $$0=\nabla \cdot S+F_{V}$$

applied to the sensor for *w* = 2 µm and *l*_C_ = 100 µm. Here *u* is the amplitude with the phase *ϕ* and *F*_V_ = *F*_tot_/*V* is the force per volume with the applied total force *F*_tot_ on the volume *V*. For the dynamic mode, a loss factor of the cantilever of *η*_s_ = 10^−5^ was added to the elastic constitutive matrix according to *D*_c_ = (1 + i*η*_s_) *D*. Both methods show similar behavior. Due to its simplicity, stationary mode was used to find the optimum *p*_S_ relative to *l*_C_ for three different widths (i.e., *w* = 2, 4, and 6 µm). A maximum first principal stress was found at *p*_S_ ≈ 0.2 × *l*_C_ from the cantilever clamped end.

Correspondingly, we found optimum values of *w* and *t* at *w*_S_ = 2 µm as shown in Figure 5b. The electrical resistance of the struts should be *R* = 1–2 kOhm, therefore, to avoid non-linearity and movement of the struts, *l*_S_ = *p*_S_ was defined. Two different cantilever sizes were fabricated; the parameters are listed in Table 2. A fundamental in-plane resonance frequency of *f*_0_ ≈ 440 kHz and very small resonator masses of 1.72 ng and 4.86 ng for *w* = 3 µm and *w* = 6 µm can be obtained, respectively. With a reasonable frequency resolution Δ*f* of 1 Hz [25], a minimum detectable mass of ~7.8 fg can be expected using Equation (1). This corresponds to a single spherical carbon particle (density of 2.6 g/cm^3^) with a diameter of ~180 nm.

## 4. Tests and Measurement Results

Sensors were fabricated using <100> *n*-type bulk silicon wafers. The electrical integration was realized by photolithography, thermal oxidation, dopant diffusions using Borofilm 100 (for *p*-type piezo resistors) and phosphorosilica (for *n*-type bulk and ground contacts) emulsions from Emulsitone Chemicals, LLC (Washington, WA, USA, http://www.emulsitone.com) and an evaporated Cr/Au layer for the contact lines (Figure 3). We assume a *p*-diffusion depth of ~1.4 µm (measured by a monitor sample using an electrochemical capacitance-voltage (ECV) profiler), which defines the piezo-resistive stress-sensing part of the struts. For etching, inductive coupled plasma (ICP) cryogenic dry etching processes were used. The cantilever structure on the front side was anisotropic etched at a temperature of −95 °C and a O_2_ flow rate of 9 sccm. Subsequently, an isotropic under-etching step was done by lowering the O_2_ flow rate to 4.5 sccm to release the resonator. The micro-channels are anisotropic etched from the back side.

**Figure 6:**
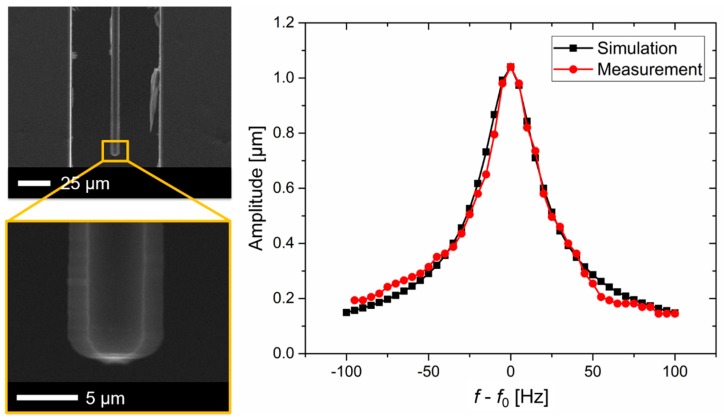
Top-view SEM images of a fabricated Si cantilever excited in in-plane (**left**) with measured and simulated displacements around the resonance frequency (**right**).

For proof-of-principle measurements in the laboratory, an etched cantilever structure was excited inside an SEM chamber using a piezo shear actuator (PICA Shear Actuator P-121.01 from Physik Instrumente (PI) GmbH & Co. KG, Karlsruhe, Germany, www.physikinstrumente.de) and a waveform generator (Hewlett Packard 33120A). By measuring the tip displacement of the cantilever by the FEM images over frequency, a resonance curve with *f*_0_ = 465 kHz was found as shown in Figure 6. A high-quality factor of *Q* = 15,500 was calculated by

(4) $$Q=\frac{f_{0}}{BW}$$

where *BW* is the band width of the resonance curve. The cantilever displacement was modeled by adapting *η_s_* = 1/*Q* to the FEM data base.

**Figure 7:**
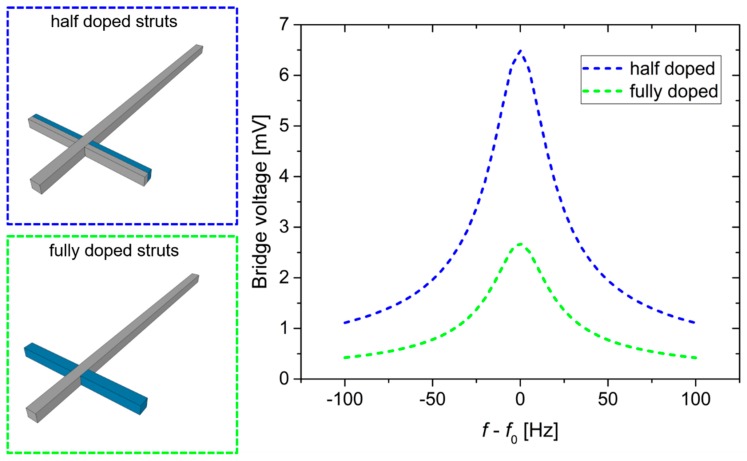
Electro-mechanical simulation of the cantilever structure with half-doped and fully doped struts.

Furthermore, the electrical behavior of the device was analyzed. By adding the measured damping parameter to the model, we obtained more reliable simulation results. Thereby, the electrical output signal of the Wheatstone bridge corresponding to the cantilever deflection in resonance was determined as shown in Figure 7. Corresponding to the doping design of the struts, two different piezo resistor configurations were simulated. In the first case, the struts are *p*-doped over their full width, which is appropriate to detect longitudinal stress, but insensitive to bending deformation. In the second case, the struts are *p*-doped over half of their width, which should not affect their sensitivity to longitudinal stress. However, strut bending should be detectable too. As expected, the half-doped struts demonstrate much higher sensitivity than the fully doped struts.

## 5. Particle Collection and Separation

**Figure 8:**
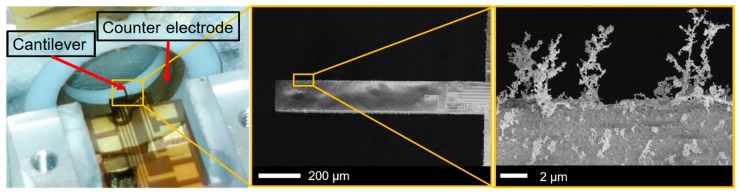
Photograph and SEM images of a cantilever resonator inside a custom-built sampler and with electrostatically collected NPs, respectively.

**Figure 9:**
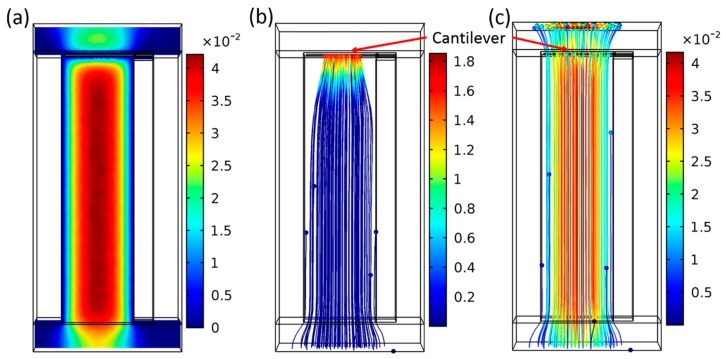
COMSOL simulation results of NP tracing in a micro channel (80 × 50 µm^2^, side view) showing (**a**) the flow velocity, (**b**) particles with 10 nm in diameter, and (**c**) particles with 2.5 µm in diameter. The colors represent the velocity (in m/s) of the air flow and of the particles. A pressure difference of 1 Pa and a negative electric potential of −100 V were applied to the collecting electrode on the cantilever.

**Figure 10:**
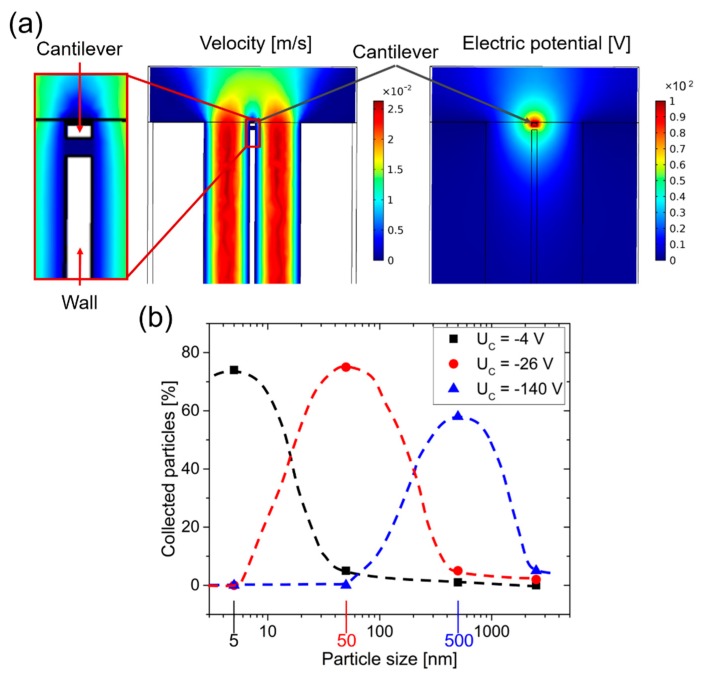
(**a**) COMSOL simulation results of NP tracing (from bottom to top) and electric potential distribution (collecting voltage = 100 V) in two adjacent micro channels (each with 100 × 25 µm^2^ cross section) at a pressure difference of 1 Pa and (**b**) sampling efficiencies in dependence on particle size (i.e., 5 nm, 50 nm, 500 nm, and 2.5 µm) at collecting voltages of −4 V, −26 V, and −140 V.

We are able to efficiently collect particles by their natural charge using electrostatic fields. This principle has already been proven in our previous works [19,20,24]. Figure 8 shows a cantilever sensor inside a homebuilt collecting device that has electrostatically collected TiO_2_ NPs on its surface. The airborne particles are collected inside a closed chamber with cleaned air as used for calibration measurement as presented in reference [14]. In this setup, the distance of cantilever and counter electrode is ~3 mm, and a collecting voltage of 300–600 V is applied for particle collection. Due to the strongly reduced distances of <35 µm and the improved design of µ-channels having a focused particle stream, an increased collection efficiency at much lower collection voltages for the new sensor design is expected. Therefore, FEM of particle tracing assuming laminar flow in a micro/channel with a cross-section of 50 × 80 µm^2^ was performed, as shown in Figure 9 (side view of the cantilever) and Figure 10 (front view of the cantilever). Figure 9a shows the air velocity (from the bottom to the top) inside the channel at a pressure difference of 1 Pa. This low-pressure gradient can be achieved by a miniature fan (like the HY10A03A from SEPA Europe GmbH) and result in a flow velocity of ~0.04 m/s along the central axis of the channel. Using the setup, in Figure 9b,c, positive charged particles with diameters of 10 nm and 2.5 µm, respectively, are led through the channel towards the cantilever and collected there induced by an electrical potential at the cantilever electrode of −100 V in respect to the grounded substrate. The simulation exhibits a particle-sampling efficiency of 85% for the 10 nm particles and a much lower collection efficiency of 10% for the 2.5 µm particles, which mostly pass the electric field and the cantilever due to their larger inertia.

Even though on one hand a low particle collection is not desired, this phenomenon (i.e., different particle sizes/masses resulting in different collection efficiencies) on the other hand can be used for particle separation mechanism. In Figure 10, a simulation is shown where positive charged carbon particles with diameter of 5 nm, 500 nm and 2.5 µm flowing through the micro-channel were considered. To increase the size-separation efficiency, we added a wall splitting the volume underneath the cantilever into two channels. The collection voltage *U*_C_ was varied from −1 V to −150 V. At too-low voltages, large particles pass through the channels without being attracted to the cantilever, while at too-high voltages small particles are trapped at the channel wall and do not reach the cantilever. Best results with respect to separation particle diameters of 5 nm, 50 nm and 500 nm were obtained using *U*_C_ = −4 V, *U*_C_ = −26 V, and *U*_C_ = −140 V, respectively, as shown in Figure 10b, which will be experimentally confirmed using the previously described setups [14]. The combination of flow rate and collection voltage allows the separation of UFP, but also proves to be a great challenge in terms of stability and reliability.

## 6. Conclusions

The sensor concept and design optimization using finite-element modelling (FEM) of a self-reading miniaturized cantilever for highly sensitive airborne NP detection have been presented. Due to the small geometry, the piezo-resistive out-reading has been realized by two supporting struts, where resistances are arranged in a Wheatstone half-bridge configuration. This design allows even further miniaturization and is not limited by electrical patterning integrated on the cantilever. An optimum strut position along the cantilever length *l*_C_ for maximum stress was found at 0.2 × *l*_C_. Furthermore, smaller width and thickness values show higher stress in the struts. Two different cantilever sizes with structure widths of *w* = 3 µm and *w* = 6 µm, cantilever lengths of 122.5 µm and 173.2 µm, and cantilever masses of 1.72 ng and 4.86 ng have been fabricated by standard photolithography-based processes, respectively. Besides, their corresponding resolutions were estimated to be 7.8 fg/Hz and 22.1 fg/Hz, respectively. A µ-channel to focus the particle stream and electrodes for electrostatic particle collection have been integrated for increasing the particle collection efficiency and for particle separation by controlling the collecting voltage, which could be shown using FEM. Proof-of-principle resonance measurements with cantilever test-structures have been conducted and analyzed by scanning electron microscopy combined with FEM for further electrical optimization. Regardless of the required further verification and measurements using more sensor samples, the presented results combining both experimental tests and simulations reveal a promising method to realize self-out-reading micro-/nano-cantilevers, which has high potential for a new generation of portable gravimetric nanoparticle sensors.

## Figures and Tables

**Table 1:** Material parameters of FEM in COMSOL Multiphysics.

Symbol	Material Property	Silicon
*E*	Youngs modulus (GPa)	170
*χ*	Poissons ratio	0.28
*ρ*	Density (kg/m^3^)	2330
*D*	Elasticity matrix (GPa)	$\left(\begin{array}{cccccc} 166 & 64 & 64 & 0 & 0 & 0\\ 64 & 166 & 64 & 0 & 0 & 0\\ 64 & 64 & 166 & 0 & 0 & 0\\ 0 & 0 & 0 & 80 & 0 & 0\\ 0 & 0 & 0 & 0 & 80 & 0\\ 0 & 0 & 0 & 0 & 0 & 80 \end{array}\right)$

**Table 2:** Dimensions of the fabricated test structures.

Parameter	Value 1	Value 2
Strut/cantilever width *w*_S_ and *w*_C_	3 µm	6 µm
Cantilever length *l*_C_	122.5 µm	173.2 µm
Strut length *l*_S_	25 µm	35 µm
Strut/cantilever thickness *t*	2–8 µm	2–8 µm
Strut position *p*_S_ (from clamped end)	25 µm	35 µm
